# How Do Economic Fluctuations Affect the Mortality of Infectious Diseases?

**DOI:** 10.3389/fpubh.2021.678213

**Published:** 2021-04-22

**Authors:** Ting-Ting Sun, Ran Tao, Chi-Wei Su, Muhammad Umar

**Affiliations:** ^1^School of Economics, Qingdao University, Qingdao, China; ^2^Qingdao Municipal Center for Disease Control and Preventation, Qingdao, China

**Keywords:** economic fluctuations, infectious diseases mortality, mixed frequency vector autoregression model, counter-cyclically, China

## Abstract

This paper uses the mixed frequency vector autoregression model to explore the impact of economic fluctuations on infectious diseases mortality (IDM) from China perspective. We find that quarterly gross domestic product (GDP) fluctuations have a negative impact on the annual IDM, indicating that the mortality of infectious diseases varies counter-cyclically with the business cycle in China. Specifically, IDM usually increases with deterioration in economic conditions, and vice versa. The empirical results are consistent with the hypothesis I derived from the theoretical analysis, which highlights that economic fluctuations can negatively affect the mortality of infectious diseases. The findings can offer revelations for the government to consider the role of economic conditions in controlling the epidemic of infectious diseases. Policymakers should adopt appropriate and effective strategies to mitigate the potential negative effects of macroeconomic downturns on the mortality of infectious diseases. In the context of the COVID-19 pandemic, these analyses further emphasize the importance of promoting economic growth, increasing public health expenditure, and preventing and controlling foreign infectious diseases.

## Introduction

The objective of this paper is to analyze the relationship between economic fluctuations and health outcomes in China from the perspective of infectious diseases. Safety of human beings has always been threatened by infectious diseases (1), which are the leading cause of death worldwide accounting for a quarter to a third of all mortality (2). Approximately one new human infectious disease emerges per 8 months on average (3). In recorded human history, the globe has suffered from many shared infectious diseases (4), such as the fourteenth century Black Death and the 1918 influenza pandemic, which has caused 25–40 million and 50–100 million deaths, respectively (5, 6). In recent years, the health burden of infectious diseases is believed to be becoming insignificant, because improvements in nutrition, sanitation, and public health policies have caused a steady decline in overall incidence and mortality (7, 8). However, the outbreak of several epidemics of infectious diseases, such as human immunodeficiency virus (HIV), severe acute respiratory syndromes (SARS), highly pathogenic avian influenza (H5N1), Middle East respiratory syndrome (MERS), still has produced serious economic impacts and health security threats (4, 9). Coronavirus disease 2019 (COVID-19) is a newly infectious disease with seemingly high transmissibility (2), which has spread globally since January 2020. The World Health Organization (WHO) reports that as of 15 February 2021, there have been over 108 million confirmed cases of COVID-19, including more than 2 million fatalities. The outbreak of COVID-19 not only causes increasing mortality rate, but it also generates negative spillovers to the real economic systems. There is a significant decline in trade throughout 2020 owing to the COVID-19 pandemic, and the globe is experiencing a new economic crisis (10). In light of these recent epidemics, it is clear that infectious diseases still play an important role in global health policy, transportation safety, and economy (7, 11). As the infectious diseases affect the ability to work and the accumulation of human capital, their outbreaks ultimately influence the economic growth (12). In turn, the impact of economic fluctuations on health outcomes can be far-reaching. These include shifts in trends in health risk such as immunization levels and utilization of health services, and differential impact on vulnerable groups. In the 1990s, countries of the former Soviet Union (FSU) and Eastern Europe have experienced a devastating economic crisis, as gross domestic product (GDP) has fallen by one-third on average (13). Concurrently, the incidence, prevalence and mortality of tuberculosis have risen markedly (14). HIV has increased from relatively low pre-crisis levels; diphtheria (15), tick-borne encephalitis (16) and leptospirosis have also occurred. Some increases in observed infectious diseases are seen following the 2008 global financial crisis, notably for influenza, HIV and indigenous malaria (17). Furthermore, diminished screening, treatment and case-management services are documented over the period 2008–2009 for the U.S. services in sexual health. During the European recession in 2009–2010, the government has cut budgets, specifically those related to infectious disease prevention services (18). Therefore, we can conclude that there is a close link between economic fluctuations and health conditions, as well as infectious diseases.

Since the economic reform in 1978, the Chinese economy has grown at a remarkable pace in the past few decades. China has established a complete infectious disease prevention, control, and biosafety system which effectively reduces the prevalence of infectious diseases (1). Nevertheless, the infectious diseases are still the major public health threat in China. The National Health Commission of the People's Republic of China reports that there are 5,806,728 notifiable infectious diseases cases and 26,374 deaths in 2020. The advance of globalization, frequent personnel exchanges, and close international trade cooperation make it possible for infectious diseases from all over the world to be imported into China (19). Since 2003, the outbreak of new infectious diseases, such as SARS, H5N1, and MERS, has revealed some shortcomings of China's infectious disease prevention system (1, 20). In 2009, a total of seven infectious diseases (hepatitis B and C, pulmonary tuberculosis, dysentery, syphilis, gonorrhea, and H1N1 influenza A) has a national reported incidence number >100,000. Three out of the seven major infectious diseases (i.e., pulmonary tuberculosis, syphilis, and gonorrhea) has a close correlation with economic growth (21). Conversely, the epidemic of infectious diseases can cause economic fluctuations. The current COVID-19's infectivity and transmission speed surpass those of the previous infectious diseases, and has affected many industries and society (22), causing unprecedented economic damages to China (23). In the first quarter of 2020, the GDP has represented a decrease of 6.8% compared to the same period in 2019. Obviously, there are certain links between economic fluctuations and the incidence and mortality of infectious diseases for China, this is worth exploring. However, previous studies have focused on the relationship between business cycles and health outcomes in developed countries, and little is known about emerging market economies. China exhibits almost all of the notable features that characterize emerging market countries (24). Moreover, China is a major contributor to the worldwide infectious disease burden because of its population size. Thus, we will explore the correlation between economic fluctuations and infectious diseases mortality (IDM) from the perspective of China.

This paper applies the time-series data over the period from 1992Q1 to 2019Q4 and the mixed frequency vector autoregression (MF-VAR) approach to conduct the empirical analysis. We obtain the negative impact of quarterly GDP fluctuations on the annual IDM, suggesting that the mortality of infectious diseases behaves counter-cyclically as it changes with the business cycle in China. More precisely, IDM tends to increase (decrease) during economic contractions (expansions). The results support the hypothesis I derived from the theoretical analysis, which highlights that economic fluctuations have a negative impact on the mortality of infectious diseases. These analyses can provide implications for the government to consider the role of economic conditions in controlling the epidemic of infectious diseases. High economic growth is associated with lower mortality and vice versa, this would offer guidance on the future impact of macroeconomic downturns for the spread of infectious diseases. It is necessary for the government to adopt appropriate and effective policies and strategies to mitigate the potential negative effects of infectious diseases. Besides, public health expenditure is an important pathway through which economic conditions affect the mortality of infectious diseases. This implies that the government should invest in the latest medical technology and continue to improve the public health-care system. Also, the prevention and control of foreign infectious diseases should be considered in the development of effective public health strategies in China.

There are several contributions of this paper. First, the previous studies mainly focus on exploring the correlation between economic fluctuations and health outcomes in high-income countries [see Budhdeo et al. (25), Gerdtham and Ruhm (26), Strumpf et al. (27), Tapia Granados (28, 29)]. However, few studies have examined how fluctuations in the economy affect health states in low- and middle-income countries. Such studies include Arroyave et al. (30) and Ruhm (31) for Columbia, Bhalotra (32) for India, Gonzalez and Quast (33, 34) for Mexico. While the extant literature has extensively demonstrated the relationship between the two variables, there is no specific study on China. Thus, we attempt to fill this gap by examining the impact of economic fluctuations on health outcomes from China perspective. Second, the existing literature has identified the cyclical changes of some cause-specific mortality (e.g., cancer, cardiovascular disease, suicide, adult and infant mortality) with economic conditions, but explorations of infectious diseases are relatively limited. However, infectious diseases are major threats to public health, and remains a leading source of human morbidity and mortality (7, 8). The study is a groundbreaking work to investigate whether the mortality of infectious diseases is pro-cyclical or counter-cyclical in China. Third, since traditional approach requires all data to have a single frequency, previous studies usually aggregate high-frequency variables, which may make statistical estimates inaccurate. In order to obtain the heterogeneous effects of high-frequency variables on low-frequency series, it is necessary to use the MF-VAR model that does not require any filtering procedures. Thus, we contribute to the literature by employing the MF-VAR model to explore the impact of quarterly economic fluctuations on annual mortality of infectious diseases.

The remainder of this study is arranged as follows: Section “Literature Review” provides an overview of the relevant studies. We illustrate the theoretical mechanism and research hypothesis in section “Theoretical Analysis and Research Hypothesis” and describe the MF-VAR model in section “The Mixed Frequency Vector Autoregression Model”. Section “Data” explains the data used in the paper. Section “Empirical Results” presents our findings, while section “Conclusions” concludes.

## Literature Review

The association between business cycle and mortality has been extensively studied. Specifically, most evidence from high-income countries indicates that the overall mortality is pro-cyclical—it increases (decreases) during economic expansions (contractions). Neumayer (35) holds that recessions lower some, but not all, mortality in the case of Germany. Tapia Granados (29) reveals that unemployment effects on general mortality are negative, the death rate increases pro-cyclically when joblessness diminishes in an economic expansion in Spain. Gerdtham and Ruhm (26) suggest that a 1% decrease in the unemployment rate is associated with growth of 0.4% in total mortality for Organization for Economic Cooperation and Development (OECD) countries. Tapia Granados (28) illustrates that general mortality and age-specific death rates in Japan tend to increase during expansions and conversely drop during recessions. Ariizumi and Schirle (36) point out that a 1% increase in the unemployment rate lowers the predicted mortality of Canadians in their 30s by nearly 2%. Toffolutti and Suhrcke (37) find that during the European Union (EU)' Great Recession, an increase of 1% in the standardized unemployment rate has resulted in a decrease of 3.4% in the all-cause-mortality. Ruhm (38) confirms that the effects of severe recessions in the U.S. appear to have a beneficial effect on mortality that is roughly twice as strong as that predicted by the elevated unemployment rates alone. Strumpf et al. (27) highlight that a 1% increase in unemployment rate in U.S. metropolitan area is associated with a decrease in overall mortality of 3.95 deaths per 100,000 person years. Spiteri and Brockdorff (39) demonstrate that in Europe, there is a statistically-significant inverted *U*-shaped relationship, whereby as income increases cardiovascular disease mortality first rise, before declining at subsequent levels of development. However, not all studies support this assertion, they find a counter-cyclical pattern with mortality rising during recessions. Gerdtham and Johannesson (40) suggest that for Swedish men, a significant counter-cyclical relationship does exist between the business cycle and the mortality risk. Economou et al. (41) argue that in the EU, there is a close positive relationship between adverse economic conditions and the mortality. Halliday (42) uses the individual-level data in the U.S., and illustrates that a 1% increase in the unemployment rate raises the probability of dying next year by 6%. Budhdeo et al. (25) conclude that the overall reduction in public investment, including health care, during times of crisis in the EU has been associated with an overall increase in mortality. Little is known about the relationship between business cycles and the mortality in low- or middle-income countries, with studies conducted yielding contradictory results. Ruhm (31) shows that Columbia's high unemployment rates are related to lower mortality, and vice versa. Bhalotra (32) finds that rural infant mortality in India is counter-cyclical, the elasticity being about−0.33. Gonzalez and Quast (33, 34) denote that while overall mortality appears to be pro-cyclical for Mexico, the relationship may vary by the level of development of states and differ for several specific causes of death. Similarly, Arroyave et al. (30) state that relationship between business cycles and mortality changes with period and age, and indicate that there is no evidence of pro-cyclical mortality in Colombia. Williams et al. (43) suggest that a rise in unemployment is associated with long-lasting deterioration of several population health outcomes in Latin America.

In addition, few studies explore the impact of economic fluctuations on the morbidity or mortality of infectious diseases. Kentikelenis et al. (44) suggest that migrants are especially vulnerable to infectious diseases during economic crises. Hunter et al. (17) find that the relationship between economic indicators (e.g., GDP per capita and unemployment rate) and infectious disease incidence is usually non-linear. Shahbazi and Khazaei (45) show that there is a higher concentration of morbidity and mortality of COVID-19 in countries with higher socioeconomic status. Goutte et al. (46) document that higher economic “precariousness indicators” such as unemployment and poverty rates, are important factors in determining COVID-19 mortality. Conversely, many studies evaluate the economic impacts of infectious diseases. Blake et al. (47) indicate that the outbreak of foot and mouth disease in the UK has larger adverse effects on GDP through reductions in tourism expenditure. Hai et al. (48) point out that SARS has significant negative impacts on China's economy. Verikios et al. (49) assert that H1N1 epidemic could have significant short-run macroeconomic effects but the size of these effects is highly dependent on the degree of inertia in the markets for physical capital and labor. Goenka et al. (50) state that there is a two-way interaction between the economy and the infectious disease. Kumar et al. (51) argue that H1N1 influenza has huge indirect impacts on the socio-economic framework; absenteeism and decreased manpower cause an economic slowdown. Verikios (2) denotes that pandemic influenza and human coronavirus lead to a global economic recession.

The existing literature has evaluated the relationship between economic status and health outcomes. More precisely, numerous studies support that mortality is pro-cyclical [e.g., (27, 33, 34, 38, 39)], while others find the opposite [e.g., (25, 32, 42)]. These studies mostly focus on high-income countries, but the evidence for the impact of economic fluctuations on health outcomes in low-income or middle-income countries is rather limited. Furthermore, although the extant literature has indicated the potential effects of economic fluctuations on overall mortality, relatively few analyses have yet to be done focusing on infectious diseases. That is, the impact of economic fluctuations on IDM has not been clearly explained. Motivated by the previous studies, we attempt to explore whether IDM is pro-cyclical or counter-cyclical in China. Additionally, conventional methods fail to fit variables of different frequencies to a model, and therefore cannot analyze the heterogeneous effects of high-frequency series on low-frequency variables. Therefore, in order to improve the accuracy of the estimated outcomes, we apply the MF-VAR model to examine the relationship between quarterly economic fluctuations (measured by GDP growth rate) and annual IDM.

## Theoretical Analysis and Research Hypothesis

Various mechanisms have been suggested to explain why mortality may respond to fluctuations in the business cycle (52). Two main explanations have been identified between economic fluctuations and health conditions. One perspective focuses on the social and psychological difficulties caused by the economic recession, which can explain that mortality behaves counter-cyclically as it moves with the business cycle. The material losses caused by unemployment, and the reduction of personal health expenditure may ultimately lead to unhealthy diets. In addition, the stress, anxiety and psychological difficulties associated with being unemployed or managing to stay in employment are also harmful to health. Mainly because the affected people usually resort to drugs and alcohol to alleviate their stress and hardship. Novo et al. (53) report that the physical and psychological conditions of 21-year-olds are worse during the recession compared to the period of economic expansion. There is substantial evidence that unemployed people suffer from deterioration in physical and mental health and well-being (54, 55). Another perspective is derived from the economic model of utility maximization (35, 37), this can explain that mortality varies pro-cyclically with the business cycle. Specifically, in this model, four main pathways can be summarized to explain that economic expansions (contractions) may have a negative (positive) impact on health conditions. First, during the economic boom, the opportunity cost of leisure time increases as more personal work and income. Less time is spent on health maintenance activities and routine physical examinations. As a result, health deteriorates, or mortality increases with improvements in economic conditions. Second, during the economic downturn, workers may benefit from lower stress levels due to reduced working hours. Conversely, the economic improvement can increase job-related pressures, and the affected people may resort to alcohol, medication and drugs, which instead leads to the deterioration of their health. Third, work-related accidents increase in periods of economic expansion. On the contrary, due to lower overall economic activity, work-related or other types of accidents (e.g., motor vehicle accidents) may decrease. Fourth, globalization and international travel have increased the ability of persons to move all across the globe. The economic recession reduces the incentives for immigration, and the reduction of imported diseases has led to a lower death rate at the destination. Therefore, we can obtain two different aspects of the impact of economic fluctuations on health outcomes.

The influence mechanism of the economic fluctuation on infectious diseases has received comparatively-less attention within the theoretical literature. Nevertheless, there is still evidence that the incidence of infectious diseases has increased significantly during the previous economic crisis and downturn (13). It is widely known that the recession during 2008-2009 has caused serious economic hardship for many governments and citizens around the globe. Some European countries have cut budgets for infectious disease control, putting infected people at risk of interruption of treatment. Workers are afraid of unemployment and do not want to take sick leave, which potentially increases the risk of disease transmission at work (56). In addition, economic choices on investment in health services can affect the disease transmission (50). Mainly because investment expenditures lead to accumulation of health capital, and thus reducing the incidence and infectivity of infectious diseases. For instance, vaccination against an infectious disease can reduce the number of susceptible individuals in the population, and lower morbidity and mortality during the epidemic period (57). However, the vaccination rate is usually limited by government budgets, which limits the capacity to control infectious diseases. Therefore, the worsening of the outcomes of infectious diseases during recession is usually due to higher infection rates and fewer treatment opportunities in poor economic environments. Although the above analyses may lack detail for specific diseases, it does enable an understanding of how economic fluctuations could lead to rises (as well as potential reductions) in infectious disease transmission risk.

Summarizing the above analyses, we put forward the following Hypotheses I and II.

Hypothesis I: IDM behaves counter-cyclically as it moves with the business cycle, suggesting that economic fluctuations have a negative impact on the mortality of infectious diseases.

Hypothesis II: IDM varies pro-cyclically with the business cycle, indicating that economic fluctuations have a positive impact on the mortality of infectious diseases.

## The Mixed Frequency Vector Autoregression Model

In order to prove that the choice of sampling frequency may cause changes in the empirical results, we introduce low- and mixed-frequency VAR models, respectively.

### Low-Frequency VAR

In the first stage, the benchmark low-frequency VAR (LF-VAR) model is constructed as Equation (1):

(1) $$\begin{array}{r} \left[\begin{array}{c} GDP_{t}\\ IDM_{t}\\ CDC_{t} \end{array}\right]=\overset{4}{\underset{k=1}{\sum }}\left[\begin{array}{c} a_{11,k}\;a_{12,k}\;a_{13,k}\\ a_{21,k}\;a_{22,k}\;a_{23,k}\\ a_{31,k}\;a_{32,k}\;a_{33,k} \end{array}\right]\;\left[\begin{array}{c} GDP_{t-k}\\ IDM_{t-k}\\ CDC_{t-k} \end{array}\right]+\left[\begin{array}{c} \varepsilon_{1t}\\ \varepsilon_{2t}\\ \varepsilon_{3t} \end{array}\right] \end{array}$$

where *GDP*_*t*_, *IDM*_*t*_, and *CDC*_*t*_ signify annual gross domestic product, infectious diseases mortality and Centers for Disease Control and Prevention, respectively. The CDC is responsible for collecting data, detecting and analyzing possible epidemics of diseases, and actively following reported cases (4). Specifically, the information platform in CDC can establish links between hospitals and public health services, which has played an important role in increasing the detection of infectious disease cases (58). Thus, we choose the numbers of CDC in health institutions as the control variable. Besides, each series is sufficiently differenced so that the covariance stationarity is satisfied (59). To capture potential seasonality, the lag length *k* is set to 4. *a*_*ij,k*_ represents the corresponding coefficients, with *i, j* = 1, 2, 3 and *k* = 1, …, 4. Then, based on Equation (1), *IDM*_*t*_ can be expressed as the following:

(2) $$\begin{array}{r} IDM_{t}=\overset{4}{\underset{k=1}{\sum }}\left[a_{21,k}\;GDP_{t-k}+a_{22,k}\;IDM_{t-k}+a_{23,k}\;CDC_{t-k}\right]+\varepsilon_{2t\;} \end{array}$$

Let *GDP*_*t*_ denote the average quarterly GDP, which can be written as *GDP*_*t*_ = (*GDP*_1*t*_ + *GDP*_2*t*_ + *GDP*_3*t*_ + *GDP*_4*t*_)/4. *GDP*_*it*_ represents the GDP at the i*-*th quarter of year t, where *i* = 1, …, 4. Equation (2) could be further extended to Equation (3):

(3) $$\begin{array}{r} IDM_{t}=\overset{4}{\underset{k=1}{\sum }}\left[a_{21,k}\left(\frac{1}{4}\overset{4}{\underset{i=1}{\sum }}GDP_{i,t-k}\right)+a_{22,k}\;IDM_{t-k}+a_{23,k}\;CDC_{t-k}\right]+\varepsilon_{2t} \end{array}$$

where *GDP*_*i,t*−*k*_ has a homogeneous impact of *a*_21,*k*_/4 on *IDM*_*t*_.

### Mixed-Frequency VAR

The LF-VAR model requires that all series have a single frequency, and aggregating high-frequency variables may lead to inaccurate statistical estimates. In view of this, Ghysels and Valkanov (60) propose that mixed data sampling regression is a more effective estimation than the classic method of aggregating all series to the least frequency sampling. In order to obtain the heterogeneous effects of high-frequency variables on low-frequency series, it is necessary to use the mixed-frequency VAR (MF-VAR) model, which is primarily designed for a small ratio of sampling frequencies (61, 62). Thus, a MF-VAR model consisting of quarterly GDP and annual IDM and CDC, can be constructed in the following way:

(4) $$\begin{array}{r} \left[\begin{array}{c} GDP_{1t}\\ GDP_{2t}\\ GDP_{3t}\\ GDP_{4t}\\ IDM_{t}\\ CDC_{t} \end{array}\right]=\overset{4}{\underset{k=1}{\sum }}\left[\begin{array}{c} a_{11,k}\;a_{12,k}\;a_{13,k}\;a_{14,k}\;a_{15,k}\;a_{16,k}\\ a_{21,k}\;a_{22,k}\;a_{23,k}\;a_{24,k}\;a_{25,k}\;a_{26,k}\\ a_{31,k}\;a_{32,k}\;a_{33,k}\;a_{34,k}\;a_{35,k}\;a_{36,k}\\ a_{41,k}\;a_{42,k}\;a_{43,k}\;a_{44,k}\;a_{45,k}\;a_{46,k}\\ a_{51,k}\;a_{52,k}\;a_{53,k}\;a_{54,k}\;a_{55,k}\;a_{56,k}\\ a_{61,k}\;a_{62,k}\;a_{63,k}\;a_{64,k}\;a_{65,k}\;a_{66,k} \end{array}\right]\;\left[\begin{array}{c} GDP_{1,t-k}\\ GDP_{2,t-k}\\ GDP_{3,t-k}\\ GDP_{4,t-k}\\ IDM_{t-k}\\ CDC_{t-k} \end{array}\right]+\left[\begin{array}{c} \varepsilon_{1t}\\ \varepsilon_{2t}\\ \varepsilon_{3t}\\ \varepsilon_{4t}\\ \varepsilon_{5t}\\ \varepsilon_{6t} \end{array}\right] \end{array}$$

where *a*_*ij,k*_ are coefficients matrices with *i, j* = 1, …, 6 and *k* = 1, …, 4, and ε_*it*_ is the interference term. The mixed data sampling regression can reduce the number of coefficients by fitting a function to the parameters of high-frequency variables (59, 63). In Equation (4), we observe that *GDP*_1*t*_, *GDP*_2*t*_, *GDP*_3*t*_, *GDP*_4*t*_ are stacked in a vector. Thus, in order to clearly express the connection between the economic fluctuations and IDM, Equation (3) can be further transformed as follows:

(5) $$\begin{array}{r} IDM_{t}=\overset{4}{\underset{k=1}{\sum }}\left[\overset{4}{\underset{j=1}{\sum }}a_{5j,k}GDP_{j,t-k}+a_{55,k}IDM_{t-k}+a_{56,k}CDC_{t-k}\right]+\varepsilon_{5t} \end{array}$$

since *a*_5*j, k*_ can take different values from each other under *j* = 1, …, 4, *GDP*_1,*t*−*k*_, *GDP*_2,*t*−*k*_, *GDP*_3,*t*−*k*_, and *GDP*_4,*t*−*k*_ are considered to have heterogeneous effects on *IDM*_*t*_. Then, this study performs impulse response analysis and forecast error variance decomposition for each model. We follow Wang et al. (63) and clearly set the Cholesky order, which is *GDP*_*t*_ → *IDM*_*t*_ → *CDC*_*t*_ in the LF-VAR model, and *GDP*_1*t*_ → *GDP*_2*t*_ → *GDP*_3*t*_ → *GDP*_4*t*_ → *IDM*_*t*_ → *CDC*_*t*_ in the MF-VAR model.

Generally, the conventional method uses temporal aggregation to process data of different frequencies. However, Silvestrini and Veredas (64) have demonstrated that if high-frequency variables are forced to be aggregated, statistical inferences will be inaccurate. In contrast, the MF-VAR model, which does not require any filtering procedures, has the unique advantages of capturing the heterogeneous effects of high-frequency series on low-frequency variables (59). With this method, we fit variables of different frequencies to a model. Therefore, we will apply the MF-VAR model to capture the impact of quarterly GDP fluctuations on the annual IDM.

## Data

This paper aims to explore the impact of economic fluctuations on the mortality of infectious diseases over the period from 1992Q1 to 2019Q4. From the perspective of GDP^1^ growth rate, the first high-growth period during 1991–1997 occurs after China's market reform starting from 1992 (65). Deng Xiaoping's Southern Tour in 1992 has stimulated further reform and open-door policies, leading to subsequent economic booms (66). We use quarterly GDP growth rate to measure economic fluctuations. The Chinese government has established a routine reporting system for selected infectious diseases in the 1950s (58). The mortality is often used as an indicator to measure the outbreak of infectious diseases (12), expressed as the number of deaths per 100,000 population per year. The notifiable reported infectious diseases mortality (IDM)^2^ decreases for the first time in 1992 to <1 death per 100,000 people per year. We notice that the substantial reduction in mortality from infectious diseases may have certain connection with China's rapid economic development during the same period. Besides, mortality may be affected by health services and investments (50, 67, 68). From the perspective of health institutions, all suspected and confirmed cases of infectious diseases seen in China's vast hospital system would be immediately reported to the Center for Disease Control and Prevention (CDC). Then, CDC will ensure that the infectious diseases case is properly diagnosed and managed, and that appropriate measures are undertaken to control (58). This shows that CDC plays a key role in reducing the infectivity of infectious diseases and improving the recovery rate. Thus, we choose the numbers of CDC^3^ in health institutions as a control variable to further explain changes in mortality from the infectious disease.

**Figure 1:**
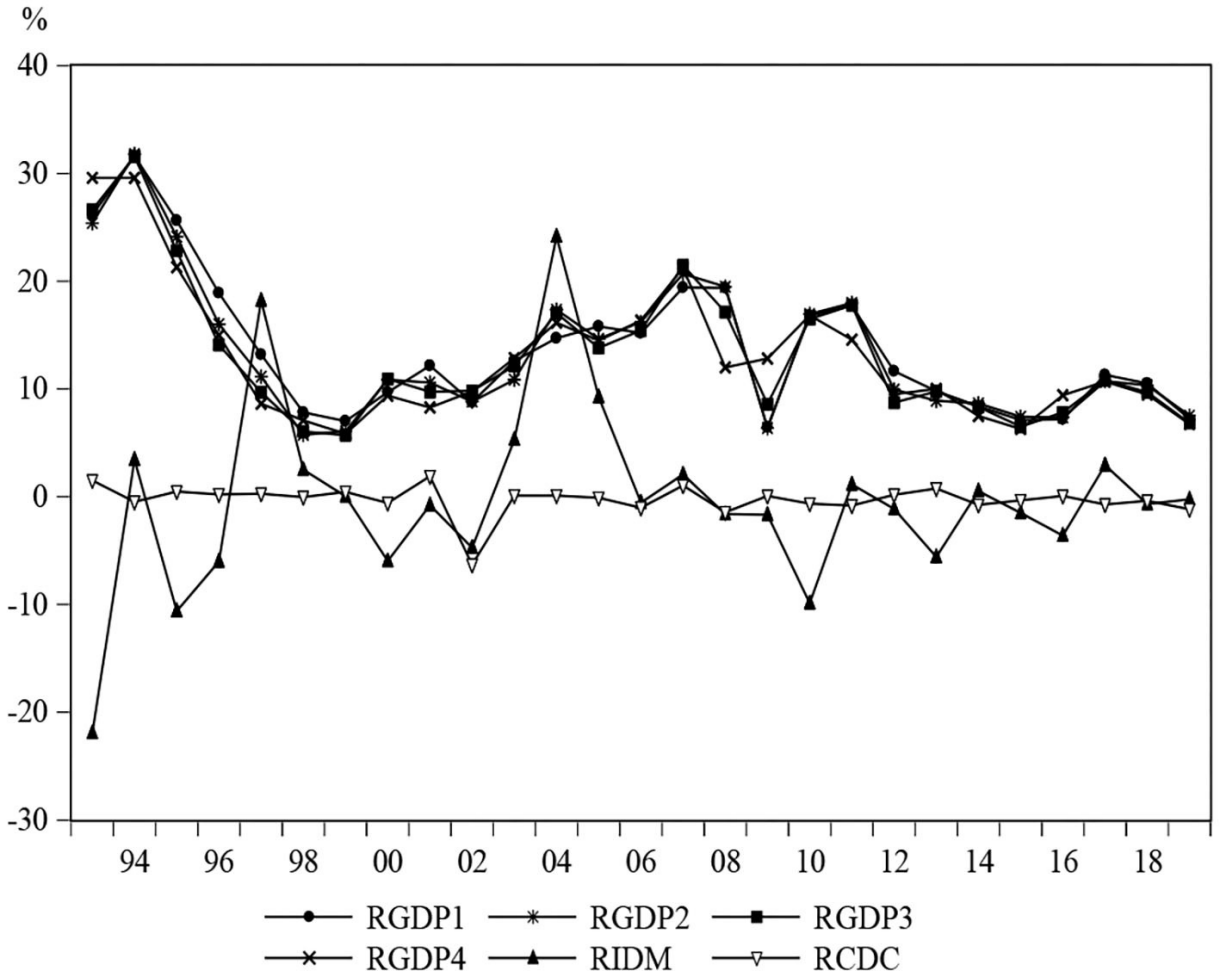
The change trends of GDP_i_, IDM and CDC. This figure presents the growth rates of GDP_1_, GDP_2_, GDP_3_, GDP_4_, IDM, and CDC from 1992 to 2019.

It can be clearly seen from the Figure 1 that 5 years with significant fluctuations in IDM are 1993, 1997, 2004, 2010, and 2017, respectively. During the high economic growth period of China's market reforms in 1993, with the improvement of living standards, public health activities have increased, and thus leading to a considerable drop in the incidence and mortality of infectious diseases. The growth rates of China's GDP have declined due to the Asian financial crisis in 1997 (69), but IDM has experienced an upward trend. The outbreak of SARS in 2003 has revealed some shortcomings of China's infectious disease prevention system (20). The incidence and mortality of infectious diseases have shown a significantly increasing trend in 2004. Meanwhile, the expansion of fixed assets investment in the whole society has triggered the second rapid growth of the Chinese economy (65). More frequent exchanges of economic activities and close business behaviors have increased the spread of infectious diseases, such as AIDS. The implementation of the 4-trillion-Yuan economic stimulus package has maintained the economic growth in 2010. During this period, public health expenditure has increased, which is conducive to the prevention and control of infectious diseases, thereby leading to a decline in IDM. China's economy has maintained a relatively stable and rapid growth especially before 2012 (70), but since then, a significant decline trend can be detected in GDP series. The slowdown in economic growth has led to the implementation of supply-side structural reforms in 2016 (71), which has strengthened the vitality of the whole society and promoted rapid economic development in 2017 (72). However, there is a slight rise in IDM; we can explain it from the perspective of the foreign infectious diseases. The number of infectious diseases among foreign cases reported in China present an increasing trend in 2017 (4), which has posed serious public health security threats. Moreover, with the development of the economy, the substantial increase in the migrant population and commercial sex has made the prevention and control of infectious diseases more difficult. Thus, we can conclude that there is a close link between economic fluctuations and the mortality of infectious diseases.

Table 1 presents the results of the descriptive statistics. The GDP growth rate series have relatively equal values in terms of mean and standard deviation. The negative skewness of CDC reflects that this variable is left-skewed distributions, but all other series are positively skewed. Since the kurtosis values of all series are >3, this suggests that they have a leptokurtic distribution. Furthermore, the Jarque-Bera index indicates that at the significance level of 1%, all these variables are non-normally distributed. However, Ghysels et al. (61) suggest that the normality assumption is not necessary for the MF-VAR model. Traditional unit root tests, such as Augmented Dickey-Fuller (ADF), Phillips-Perron (PP), and Kwiatkowski-Phillips-Schmidt-Shin (KPSS) tests (73–75), do not consider structural breaks in detecting process. Thus, Enders and Lee (76) add flexible Fourier transform into a unit root detection process, which can well-recognize unknown breaks without considering their dates and locations. Table 2 reports the corresponding results, and all series are stationary at the 1% significance level; also, the spurious connection among GDP, IDM and CDC is excluded. Therefore, these three variables can be used in the LF-VAR and MF-VAR models.

**Table 1:** Descriptive statistics.

	**GDP_**1**_**	**GDP_**2**_**	**GDP_**3**_**	**GDP_**4**_**	**GDP_**A**_**	**IDM**	**CDC**
Mean	0.138	0.134	0.132	0.130	0.133	−0.003	−0.003
Median	0.122	0.108	0.108	0.107	0.107	−0.007	0.000
Maximum	0.316	0.318	0.316	0.296	0.311	0.241	0.019
Minimum	0.064	0.057	0.057	0.058	0.062	−0.219	−0.063
Std. Dev.	0.065	0.066	0.065	0.064	0.064	0.085	0.014
Skewness	1.060	1.045	1.190	1.316	1.184	0.576	−2.676
Kurtosis	3.536	3.484	3.852	4.180	3.809	5.425	12.899
Jarque-Bera	5.382^***^	5.178^***^	7.192^***^	9.364^***^	7.063^***^	8.108^***^	142.460^***^

*This table reports the descriptive statistical summary for the growth rate of each variable. GDP_A_ denotes the aggregate average of the growth rates of GDP_1_, GDP_2_, GDP_3_, and GDP_4_*.

*** *indicates the statistical significance at 1%*.

**Table 2:** Statistics of fourier unit root test.

**GDP_**1**_**	**GDP_**2**_**	**GDP_**3**_**	**GDP_**4**_**	**GDP**	**IDM**	**CDC**
−7.622^***^	−6.358^***^	−6.275^***^	−7.831^***^	−6.193^***^	−12.364^***^	−10.085^***^

*** *denotes the statistical significance at 1%. The critical values for the statistics are taken from Enders and Lee (76)*.

## Empirical Results

**Figure 2:**
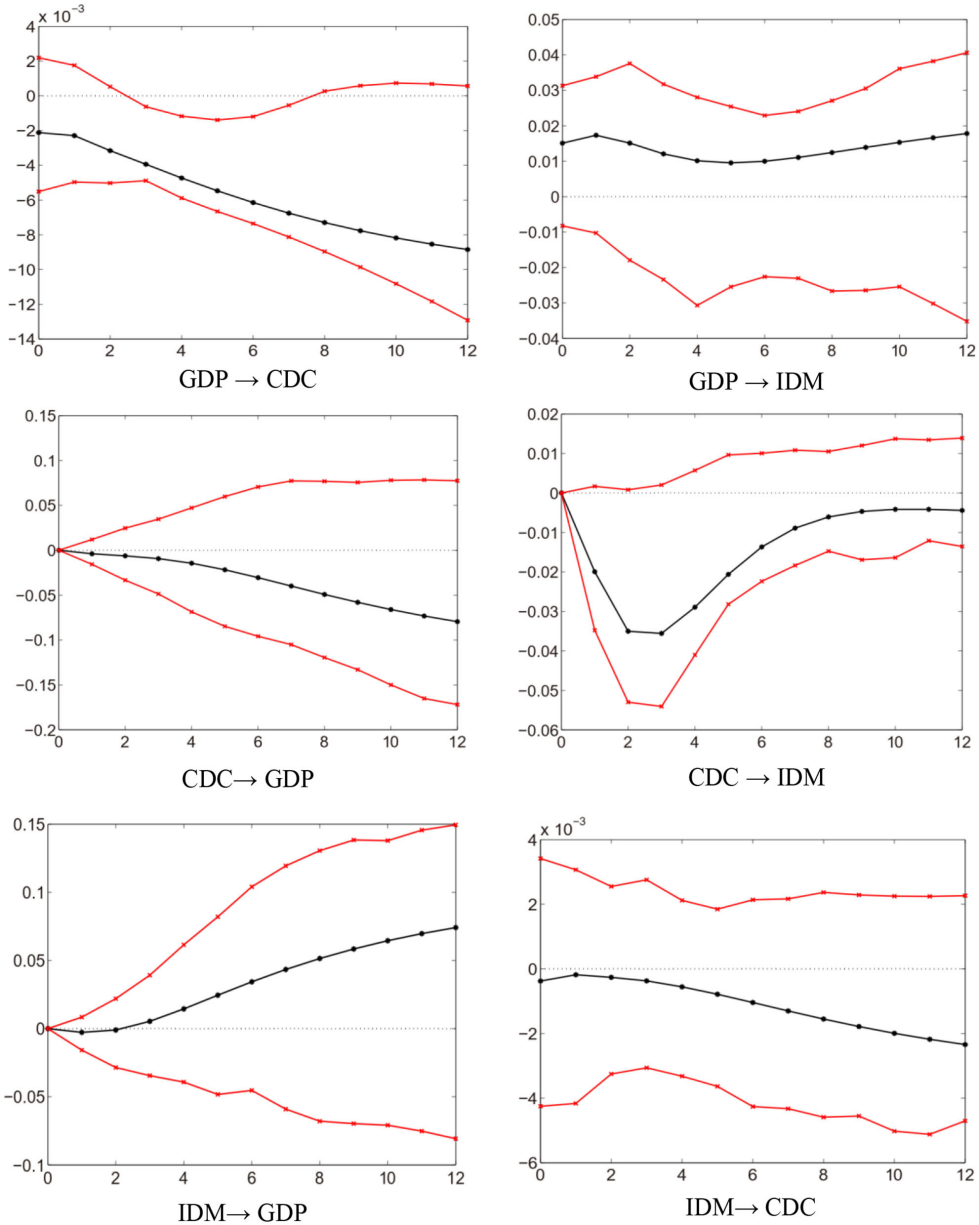
Impulse response functions of LF-VAR (4).

In the first stage, we analyze the empirical results of the LF-VAR model. The impulse response function reported in Figure 2 has a 95% confidence interval, which is constructed by the parameter bootstrap (with a sample frequency of 10,000) at each horizon h = 0, …,12. We notice that there is no significant impact from GDP to IDM. However, the impulse response of IDM to a 1 shock from CDC (expressed as “CDC → IDM”) is negative at the horizon h = 0, …,12. We can explain it from two sides. First, the National Notifiable Infectious Disease Reporting Information System (NNIDRIS) is currently the largest system for reporting infectious disease cases in China, to help collect information on disease occurrence and epidemics, and provides an early warning for outbreaks (4, 77). The CDC at all levels across the country can obtain information about infectious diseases in real time from NNIDRIS, and carry out prevention and control in advance, thereby effectively reducing the risk of infectious disease epidemics. Second, all suspected and confirmed cases of infectious diseases seen in China's vast hospital system would be immediately reported to the local CDC. Then, CDC staffs will ensure that the infectious diseases case is properly diagnosed and managed, and that appropriate measures are undertaken to control (58). Thus, the negative impact of CDC on IDM can be evidenced. As the number of CDC increases (decreases), IDM tends to decline (rise). We can conclude that in China, CDC plays an important role in controlling the mortality of infectious diseases.

In Table 3, the forecast error variance decomposition of IDM in the LF-VAR model confirms the large explanatory power of CDC, and the small explanatory power of GDP. In the long-run h = 12, the forecast error variance of IDM is explained 13.6% by GDP, 38.4% by CDC, and 48.0% by itself. Overall, the LF-VAR model exhibits a relatively weak degree of interdependence across variables. Thus, the LF-VAR model fails to explain the changes in IDM from the perspective of economic fluctuations. This is because the GDP series is an aggregated high-frequency variable, which causes inaccurate statistical inferences. In view of this, we will employ the MF-VAR model to revisit the correlation between GDP and IDM.

**Table 3:** Forecast error variance decomposition of LF-VAR (4).

	**GDP**	**IDM**	**CDC**
**Decomposition of GDP**			
h = 4	0.995	0.001	0.004
h = 8	0.961	0.021	0.018
h = 12	0.918	0.041	0.041
**Decomposition of IDM**			
h = 4	0.070	0.605	0.325
h = 8	0.088	0.516	0.396
h = 12	0.136	0.480	0.384
**Decomposition of CDC**			
h = 4	0.182	0.002	0.816
h = 8	0.203	0.012	0.785
h = 12	0.181	0.030	0.789

*The model is LF-VAR (4) of yearly GDP, IDM, CDC, and we perform the forecast error variance decomposition of each series at prediction horizons h = 4, 8, and 12 years*.

**Figure 3:**
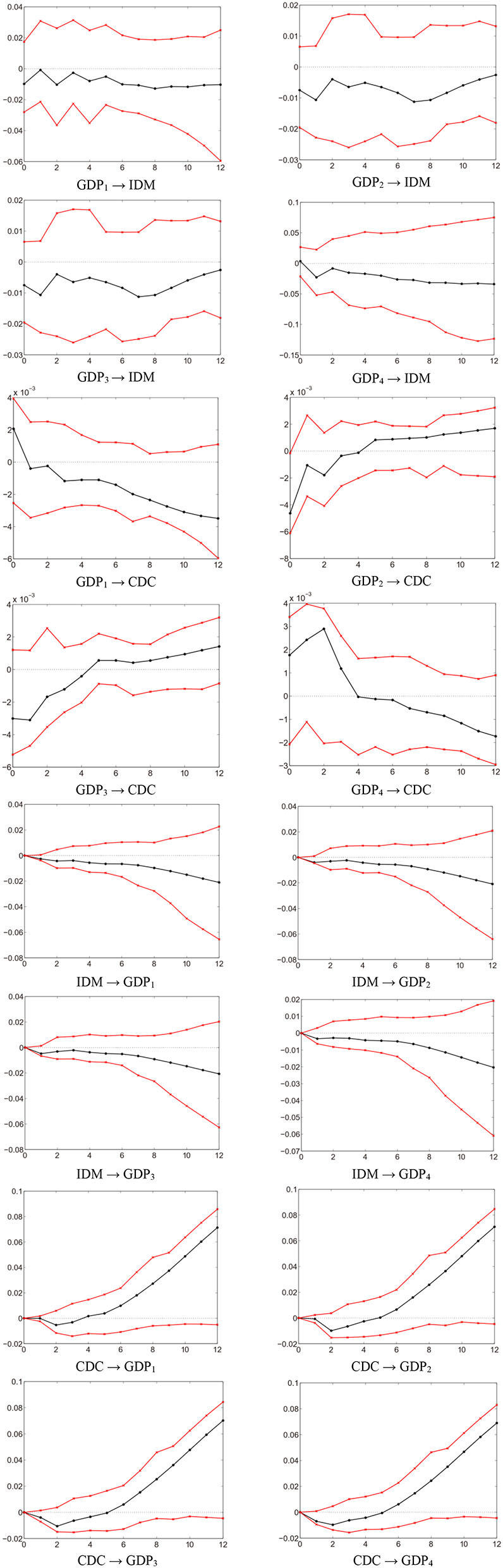
Impulse response functions based on MF-VAR (4).

The forecast error variance decomposition of the MF-VAR model is shown in Table 4. We note that the GDP series have a greater explanatory power for IDM. The long-term forecast error variance of IDM is attributed to GDP_*i*=1,2,3,4_ by 67.3%, CDC by 13.9%, and IDM itself by 18.8%. The total contribution of the GDP series is as large as 67.3% as opposed to 13.6% in the LF-VAR model. This suggests that aggregating quarterly GDP into an annual level underestimates the impact of economic fluctuations. Figure 3 presents the impulse response functions based on the MF-VAR model. GDP → IDM is significantly negative at any horizon, indicating that the mortality of infectious diseases behaves counter-cyclically as it changes with the business cycle in China. More precisely, this finding demonstrates that IDM tends to increase (decrease) during economic contractions (expansions).

**Table 4:** Forecast error variance decomposition of MF-VAR (4).

	**GDP_**1**_**	**GDP_**2**_**	**GDP_**3**_**	**GDP_**4**_**	**Sum (GDP_**i**_)**	**IDM**	**CDC**
**Decomposition of GDP**_**1**_							
h = 4	0.727	0.095	0.007	0.157	0.986	0.008	0.007
h = 8	0.848	0.037	0.009	0.077	0.971	0.009	0.021
h = 12	0.685	0.085	0.039	0.073	0.882	0.012	0.106
**Decomposition of GDP**_**2**_							
h = 4	0.657	0.151	0.012	0.149	0.969	0.006	0.025
h = 8	0.840	0.056	0.009	0.067	0.972	0.007	0.021
h = 12	0.680	0.093	0.040	0.069	0.882	0.012	0.107
**Decomposition of GDP**_**3**_							
h = 4	0.642	0.183	0.023	0.114	0.962	0.007	0.031
h = 8	0.836	0.067	0.012	0.057	0.972	0.007	0.022
h = 12	0.677	0.097	0.040	0.066	0.880	0.012	0.108
**Decomposition of GDP**_**4**_							
h = 4	0.635	0.190	0.020	0.116	0.961	0.005	0.035
h = 8	0.833	0.070	0.011	0.057	0.971	0.007	0.023
h = 12	0.678	0.098	0.040	0.066	0.882	0.012	0.107
**Decomposition of IDM**							
h = 4	0.190	0.129	0.058	0.187	0.564	0.266	0.170
h = 8	0.176	0.150	0.068	0.239	0.633	0.221	0.146
h = 12	0.177	0.162	0.081	0.253	0.673	0.188	0.139
**Decomposition of CDC**							
h = 4	0.038	0.167	0.149	0.121	0.475	0.002	0.523
h = 8	0.085	0.169	0.143	0.114	0.511	0.003	0.486
h = 12	0.210	0.153	0.119	0.105	0.587	0.008	0.405

*The model is MF-VAR (4) of quarterly GDPi, annual IDM and CDC, and we perform the forecast error variance decomposition of each series at prediction horizons h = 4, 8, and 12 years*.

There are several ways to explain that high economic growth is associated with lower IDM and vice versa. First, economic choices on investment in health services can affect the disease transmission (50). Generally, public health expenditure decreases when the economy contracts and increases when the economy expands. Public investment expenditure affects the incidence and infectiousness of infectious diseases through the accumulation of health capital. For instance, routine vaccination nationwide is considered to be the most effective way to combat the outbreak of infectious diseases or curb a disease from spreading further (57). However, the vaccination rate is usually limited by government budgets, which affects the capacity to control infectious diseases. The first high-growth period during 1991–1997 occurs after China's market reform starting from 1992 (65). With the improvement of the economic level and the increase of public health expenditure, the incidence and mortality of infectious diseases have declined to varying degrees. Subsequently, the growth rate of Chinese economy has declined due to the Asian financial crisis in 1997, government health expenditure as a percentage of GDP has fallen to 0.66%. However, IDM experiences an upward trend, with a growth rate of 26.45%. This evidences that the negative impact of economic conditions on health outcomes; IDM usually increases with deterioration in economic conditions, and vice versa. Second, from the perspective of several significant economic fluctuations in China, the counter-cyclical changes in the mortality of infectious diseases can also be captured. For instance, affected by the global financial crisis, the outbreaks of pulmonary tuberculosis, syphilis, and gonorrhea in China in 2009 are closely related to the economic recession (21). Chinese economy has entered an economic recession at the business cycle frequency since about 2014 (66), and a significant decline trend can be detected in GDP series. In the context of the economic downturn, the notifiable reported IDM has shown a continuous upward trend from 2014 to 2019; with rates rising from 1.19 per 100,000 to 1.79 per 100,000. In 2019, China's economy has experienced a significant decline due to the global trade war, especially the Sino-U.S. trade friction (78). The outbreak of COVID-19 in Wuhan in December 2019 has further strengthened the negative impact of economic fluctuations on health conditions. Third, economic fluctuations have an effect on the living burden and health conditions of individuals. During the economic recession, the unemployed suffer from the deterioration of physical and mental health and well-being (54, 55). Specifically, the material losses caused by unemployment, and the reduction of personal health expenditure may ultimately lead to unhealthy diets. In addition, the stress, anxiety and psychological difficulties associated with being unemployed or managing to stay in employment are also harmful to health. This explanatory has identified the effect of economic fluctuations on overall health conditions. It further enables an understanding of the counter-cyclical pattern with mortality rates rising during the economic recession. Fourth, the foreign infectious disease is considered as a major threat to Chinese public health. Foreign visitors to China have become more common, especially in recent years. The number of infectious diseases among foreign cases reported in China has showed an increasing trend between 2004 and 2017 from 517 to 7,958, with an average annual increase of 23.40% (4). The opportunities for foreigner cases to access to health services in China, their cultural mentalities and individual behavior have a close connection with the risk and burden of infectious diseases. The economic downturn may limit the ability to prevent and control foreign infectious diseases, which leads to a negative correlation between economic conditions and mortality. Therefore, the above analysis clearly explains that the mortality of infectious diseases varies counter-cyclically with the business cycle in China.

To sum up, we compare the LF-VAR and the MF-VAR models, in order to highlight that changing the sampling frequency can alter empirical results considerably. We find that the LF-VAR model fails to explain the changes in IDM from the perspective of economic fluctuations. However, the GDP series has a greater explanatory power for IDM in the MF-VAR model. Besides, IDM is negatively associated with GDP, suggesting that the mortality of infectious diseases behaves counter-cyclically as it changes with the business cycle in China. More precisely, this finding demonstrates that IDM tends to increase (decrease) during economic contractions (expansions). The results support the hypothesis I derived from the theoretical analysis, which highlights that economic fluctuations have a negative impact on the mortality of infectious diseases.

## Conclusions

This paper investigates the relationship between economic fluctuations and health outcomes in China from the perspective of infectious diseases. In order to obtain the heterogeneous impact of quarterly GDP fluctuations on the annual IDM, we apply the MF-VAR model that does not require any filtering procedures. Compared with the LF-VAR model, quarterly GDP fluctuations have a greater explanatory power for the annual IDM in the MF-VAR model. This indicates that aggregating quarterly GDP into an annual level in the LF-VAR model underestimates the impact of economic fluctuations. In addition, we find that GDP fluctuations have a negative impact of on IDM, suggesting that the mortality of infectious diseases varies counter-cyclically with the business cycle in China. Specifically, IDM usually increases with deterioration in economic conditions, and vice versa. The findings coincide with the hypothesis I derived from the theoretical analysis, which suggests that economic fluctuations can negatively affect the mortality of infectious diseases.

Understanding the relationship between economic fluctuations and the mortality of infectious diseases has implications for China and other developing countries. First, high economic growth is associated with lower mortality and vice versa. This finding can help inform policymakers regarding the role of economic fluctuations in managing pandemics. Specifically, the differential responses of infectious diseases to economic conditions should be taken into consideration in the development of effective public health strategies in China. Besides, it would provide guidance on the future impact of macroeconomic downturns for the spread of infectious diseases. It is necessary for the government to adopt appropriate and effective policies and strategies, which can not only mitigate the potential negative effects of infectious diseases, but also maximize the efficiency of the health system during the economic downturn. Second, public health expenditure is an important pathway through which economic conditions affect the mortality of infectious diseases. This implies that developing countries including China should invest in the latest medical technology and continue to improve the public health-care system. In the context of the global COVID-19 outbreaks, while the government strives to promote economic growth, it must ensure that CDC plays a key role in preventing and controlling infectious diseases. Third, frequent exchanges and close economic connections around the globe have increased the risk of foreign infectious diseases spreading to China or other countries. In particular, governments in developing countries should consider strengthening border inspections, developing international cooperation and early warning as effective measures to prevent and control the epidemics of infectious diseases. The correlation between economic fluctuations and health outcomes could be a fruitful area for future study. We intend to examine the relationship between the level of economic development in different regions of China and the incidence of infectious diseases in future studies; thus, a new method (e.g., panel threshold regression model) will be utilized.

## Data Availability Statement

The original contributions presented in the study are included in the article/supplementary material, further inquiries can be directed to the corresponding author/s.

## Author Contributions

T-TS: conceptualization, methodology, and software. RT: data curation and writing-original draft preparation. C-WS: visualization and investigation. MU: writing-reviewing and editing. All authors contributed to the article and approved the submitted version.

## Conflict of Interest

The authors declare that the research was conducted in the absence of any commercial or financial relationships that could be construed as a potential conflict of interest.

## Footnotes

^1^GDP is obtained at a quarterly frequency from the National Bureau of Statistics of China.

^2^IDM is taken from the Wind database.

^3^CDC is obtained from the Wind database.
